# MUC5B modulation of early oral biofilm glucose metabolism

**DOI:** 10.3389/froh.2025.1516025

**Published:** 2025-02-11

**Authors:** Carolina Robertsson, Julia Davies, Gunnel Svensäter, Anders Bay Nord, Niclas Norrström, Claes Wickström

**Affiliations:** ^1^ Department of Oral Biology and Pathology, Faculty of Odontology, Malmö University, Malmö, Sweden; ^2^ Biofilms Research Center for Biointerfaces, Faculty of Health and Society, Malmö University, Malmö, Sweden; ^3^ Swedish NMR Centre, Gothenburg University, Gothenburg, Sweden; ^4^ Department of Biology and Bioinformatics, School of Bioscience, University of Skövde, Skövde, Sweden

**Keywords:** salivary mucin MUC5B, oral biofilms, bacterial glucose metabolism, oral microbiology, *Streptococcus*, *Actinomyces*

## Abstract

**Introduction:**

Salivary mucin MUC5B has been suggested to support eubiosis in early oral biofilms by regulating the attachment of commensals, while downregulating dysbiotic activities related to dental caries development, such as microbial carbohydrate transport and metabolism.

**Methods:**

To investigate how the metabolism of glucose, a potential driver for dental caries, in early mono- and dual-species biofilms of oral *Actinomyces naeslundii* and *Streptococcus gordonii* clinical isolates was affected by the presence of the complex salivary mucin MUC5B, this study employed nuclear magnetic resonance (NMR)-based metabolomics with the interpretation of network integration.

**Results and discussion:**

MUC5B reduced early attachment in the presence of glucose compared with uncoated surfaces but maintained even species distribution. This suggests that MUC5B may represent an innate mechanism to regulate biofilm eubiosis by supporting early coadhesion while regulating total biomass. All annotated metabolites were intermediates in either carbohydrate metabolism, pyruvate conversion, or amino acid metabolism, which was not unexpected in biofilm glucose metabolomes from two saccharolytic species since pyruvate conversion represents a junction point between glycolysis and amino acid metabolic chains. The 10 metabolites present in all early biofilms represent a core metabolome shared by *A. naeslundii* and *S. gordonii*. Such core metabolomes can be used to detect deviations in future studies. Significant differences in metabolite abundance elicited by the presence of MUC5B were also detected. In early biofilms where they were each present, pyruvate, ethanol, and metabolite 134 were present in significantly higher abundance in the presence of 25% MUC5B with 20 mM glucose (MUC5B + G) compared with a physiologic buffer with 20 mM glucose (PBS + G), while metabolites 84, 97, and sarcosine were present at significantly lower abundance. Metabolite 72 was unique to biofilms grown in MUC5B + G, and eight unannotated metabolites were unique to biofilms grown in PBS + G. A pathway enrichment analysis of the metabolites that were differently expressed in early *A. naeslundii*, *S. gordonii*, and dual-species biofilms grown with 20 mM glucose with or without MUC5B showed that pyruvate metabolism was significantly over-represented. Studying the metabolic interactions between commensal members of oral biofilms and modulatory effects of host factors such as glycoproteins in saliva during the metabolism of substrates that are potential drivers of dysbiosis, such as glucose, is essential to understand the roles of oral microbial ecosystems in oral health and disease.

## Background

In their Global Oral Health status report for 2022 (1), the World Health Organization reported that oral diseases affect an estimated 3.5 billion people worldwide. The oral health conditions that represent the highest global burden are the two bacterial biofilm–induced diseases, dental caries and periodontal disease, and untreated dental caries in permanent teeth is the most common noncommunicable disease overall (2). Dental caries and periodontal disease may impact the quality of life of those who suffer from it to various degrees, through functional, psychologic, and/or social effects or pain (3, 4). In addition, the costs associated with oral disease are high (5, 6), costing over 25 billion kronor in Sweden in 2019 (6). Improving the prediction and prevention of oral biofilm–induced disease would thus reduce both suffering and costs, as well as benefit the environment by reducing the utilization of dental materials.

The mouth is a habitat for several hundred bacterial species, existing in planktonic form in saliva and as complex biofilm communities attached to the oral surfaces (7). The commensal microflora can be divided into early and late colonizers, where the cellular activities of early colonizers modify the biofilm environment so that later colonizers can establish themselves (8, 9). The metabolic networks in oral biofilms are complex (10, 11), involving both intra- and interspecies interactions as well as interplay with components from host salivary and gingival crevicular fluid, which constitute the main sources of nutrients for oral bacteria (12, 13). Many oral bacteria are saccharolytic and possess cellular machinery to readily take up large amounts of glucose from the host diet (as a free monosaccharide or scavenged from sucrose or other carbohydrates) (14–16). High concentrations of glucose in the local environment shift the metabolic output of commensal species, e.g., *Actinomyces*, *Lactobacillus*, and *Streptococcus*, toward stronger organic acids such as lactate (8, 16, 17). When the local environment in the biofilm becomes more acidic, phenotypic changes are elicited in some biofilm bacteria, which leads to a selection of saccharolytic, aciduric species, which, in turn, continue to drive the conditions in the biofilms toward dysbiosis (8), leading to the dissolution of mineralized tooth surfaces and the formation of cavities in the dental hard tissues. Once the host barrier is breached, the carious biofilms stratify, mature, and manifest in these cavities (10). Over time, there is a risk that the oral bacteria penetrate into the pulp, infect underlying soft tissues, and possibly spread into the bloodstream and cause opportunistic infections elsewhere in the body (4, 10, 16).

*Actinomyces* and *Streptococcus* are two of the most common genera isolated from oral biofilms overall (18). Both of these genera contain species that can switch between phenotypes that may contribute to driving oral biofilm dysbiosis to various degrees (7, 8). Despite the large metabolic resemblance between these two saccharolytic genera, their species and strains also differ in some respects, such as in enzymes, for transport and metabolism of carbohydrates (14, 19). When members of oral biofilms such as *Actinomyces* and *Streptococcus* species coaggregate and cooperate, these organisms can take advantage of each other's metabolic capabilities to increase their competitiveness in dental plaque (11). In the current paper, mono- and dual-species biofilms of clinical isolates *Actinomyces naeslundii* CW and *Streptococcus gordonii* CW from dental biofilms were studied. These species represent early oral colonizers and are commensals in dental biofilms during eubiosis; however, they are also able to transition to more pathogenic, dysbiosis-related phenotypes in response to certain external cues such as glucose- or acid challenge and can be present on both healthy and carious tooth surfaces (20, 21). To understand more about oral biofilm physiology, it is of interest to study how some external cues seem to induce phenotypic transition toward dysbiosis, while others promote the eubiosis-related phenotype in these species.

Saliva contains numerous biologically functional components (22, 23). One multifunctional salivary component that has been suggested to support oral biofilm eubiosis is the salivary mucin MUC5B (24–27). MUC5B is a large multimeric and highly glycosylated glycoprotein that belongs to the mucin family and is present on many mucosal surfaces in the body (28). On all of these surfaces, MUC5B, just like other mucins, acts as a part of the innate immune system as a mechanical, chemical, and biological barrier to the surrounding world (27, 29). MUC5B also modulates microbial activity through selective attachment and provision of nutrients of oligosaccharide and polypeptide origin for oral bacteria (26, 27). In the oral cavity, MUC5B is secreted with the saliva and is present in the liquid phase as well as in the salivary film that covers all the oral surfaces, the so-called salivary pellicle (28). Previous studies suggest that the complexity of MUC5B, with its highly glycosylated protein backbone and very large size, promotes a synergistic “mucolytic” activity in oral biofilms (27, 30). Multispecies consortia or mixed biofilms from plaque samples cooperate and show increased capacity to digest mucins (30). These synergistic interactions promote biodiversity in phenotypes of commensal species and tip the oral biofilm activities from dysbiotic (e.g., biofilm acidification), which predisposes for dental caries, toward eubiotic activities (e.g., coadhesion of oral commensals and a metabolic shift toward reduced acid production), which predisposes for dental biofilm eubiosis or health (25, 26, 30). However, the mechanisms of these interactions are not yet known.

Previously, MUC5B was found to elicit synergistic effects in early biofilms of oral colonizers *S. gordonii* and *A. naeslundii* that seemed to promote attachment, while downregulating dysbiotic activities related to dental caries development (26). In this paper, mono- and dual-species biofilms of clinical isolates of *A. naeslundii* and *S. gordonii* from dental biofilms were studied to investigate whether these effects persist during glucose challenge of the biofilms. Understanding salivary-mediated regulation of early dental biofilms during external challenge of potentially dysbiotic factors is essential to better understand oral biofilm physiology and may contribute to the development of novel or improved strategies to prevent oral biofilm–induced diseases such as dental caries. The novelty in the approach of this work lies in the focus on cooperation between the host and the microbial commensal flora to promote oral health, rather than exploring the role of bacteria in the development of disease.

## Materials and methods

### Human salivary MUC5B enrichment

Collection of human saliva was approved by the Malmö University Board of Ethical Review in Research, Dnr OD 2013/11, and the Swedish Ethical Review Authority, Dnr 2023-03757-01. All samples were deposited unmarked and pooled, and no analyses were performed wherein data could be traced back to any one individual. The donating group consisted of both men and women. Whole saliva (nonstimulated) from nine healthy adults was collected on ice and pooled. For MUC5B enrichment, isopycnic density gradient centrifugation was carried out as previously described (31). Briefly, the pooled saliva was diluted 1:2 in 0.2 M NaCl and centrifuged to remove debris at 4,400 × g, 4°C for 30 min (Beckman Coulter’s Avanti J-E Centrifuge, JA 20 Rotor, CA, USA). After centrifugation, CsCl was added to set the starting density to 1.45 g/mL, and isopycnic ultracentrifugation at 36,000 rpm, 15°C for 96 h was performed to separate the salivary fractions (Beckman Coulter Optima LE-80 K Ultracentrifuge, 50.2 Ti Rotor, CA, USA). Twenty-four fractions (1.7 mL) were then collected and pooled separately. Enzyme-linked immunosorbent assays using antibodies for the MUC5B polypeptide backbone central domain (6F10-E4, Novus Biologicals, CO, USA) were utilized to detect enriched fractions, which were pooled to produce an MUC5B-enriched solution. Thereafter, the solution was dialyzed in 100 kDa molecular-weight cutoff tubing (Spectra/Por™ Dialysis Membrane Biotech CE Tubing, Thermo Fischer Scientific, Europe) against sterile 10 mM phosphate buffer with 0.07 mM NaCl pH 7.5 (PBS) and stored at −80°C until use. Protein concentration was 0.3 mg/mL, determined by freeze drying and weighing after dialysis against water for removal of salts.

### Bacterial strains

**Figure 1:**
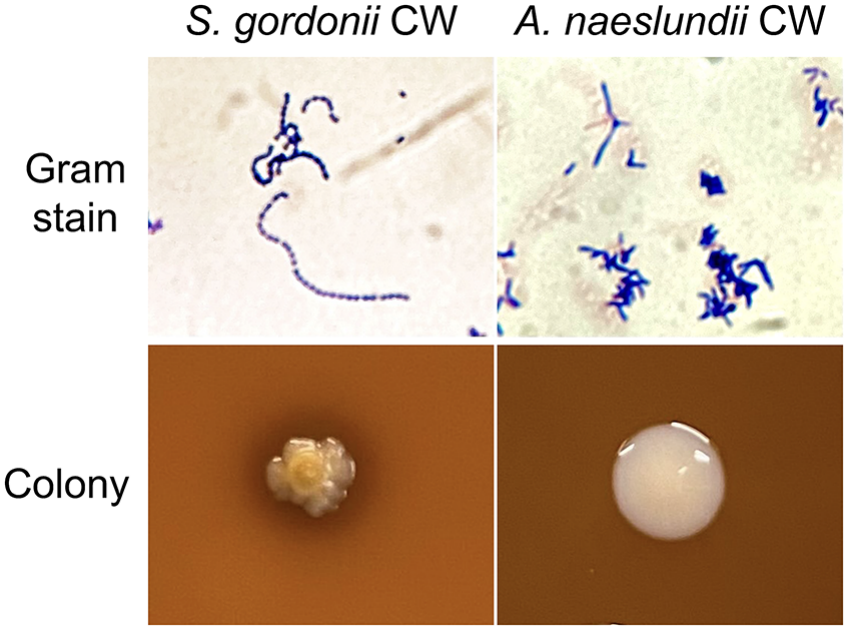
*A. naeslundii* CW and *S. gordonii* CW Gram stains and colony morphology on blood agar.

This study was based on clinical bacterial isolates *A. naeslundii* CW and *S. gordonii* CW employed in previous studies (26, 32, 33), isolated from supragingival dental biofilm of a healthy adult. Strains were selected from agar cultures based on morphology and routine phenotypic characterization. Gram stain and colony morphology of these strains is shown in Figure 1. Identification with 16S rRNA polymerase chain reaction (PCR) and Sanger sequencing (GenBank accession numbers OQ625896 for *A. naeslundii* CW and OQ625895 for *S. gordonii* CW) and whole-genome sequencing with next-generation Illumina high-throughput sequencing (GenBank accession numbers CP113787 for *A. naeslundii* CW and CP113953 for *S. gordonii* CW) were also previously performed.

### Biofilm cultures

*A. naeslundii* CW and *S. gordonii* CW from blood agar were inoculated into 25% Todd–Hewitt Yeast Extract (¼ THYE, Becton Dickinson, Denmark) and cultured overnight at 37°C in 5% CO_2_. The following day, the bacterial suspensions were washed by centrifugation (50 mL tubes, 5°C, 3,000 rpm, 10 min, Beckman GS-6R centrifuge) and resuspended in sterile PBS to an optical density (600 nm) of 0.5, which corresponds to similar cell counts between the two species, 1 × 10^8^ CFU/mL for *A. naeslundii* CW and 1.5 × 10^8^ CFU/mL for *S. gordonii* CW). The MUC5B-conditioning solution was prepared by diluting MUC5B-solution 1:5 in PBS and adding 0.1% w/v CaCl_2_ (final concentration). Twelve-well microtiter plates and Ibidi VI 0.1 μm-slides (Ibidi GmbH, Munich, Germany) were precoated with the MUC5B-conditioning solution and incubated overnight in room temperature (RT). To initiate biofilm formation, uncoated and MUC5B-coated wells of microtiter plates and Ibidi VI slides were inoculated with the planktonic *A. naeslundii* CW and *S. gordonii* CW bacterial suspensions, separately for monospecies biofilms or 1:1 v/v of each strain for dual species, to produce a final volume of 2 mL per biofilm in the wells and 100 μL in the Ibidi VI slides respectively, and incubated at 37°C in 5% CO_2_ for 2 h to allow the cells to attach. After the attachment phase, buffer supernatant and nonadherent cells were removed by gentle rinsing with PBS and replaced with 25% MUC5B in PBS with 20 mM glucose (MUC5B + G) or PBS with 20 mM glucose (PBS + G) and incubated for another 2 h as described in previous steps.

### Biofilm viability, species distribution, and biomass

Viability and species distribution were examined after incubation with MUC5B + G or PBS + G in Ibidi VI-slides in three independent *A. naeslundii* CW and *S. gordonii* CW mono- and dual-species biofilm replicates. To assess viability, the BacLight LIVE/DEAD viability kit (Invitrogen, Carlsbad, CA) was used to stain the biofilms, and imaging was performed at 60× magnification in a Nikon Eclipse TE2000-inverted confocal laser scanning microscope (CLSM) (Nikon, Japan). Illumination was done with an argon laser (488 nm laser excitation) equipped with long-pass 515/30 (green fluorescence) and 605/75 (red fluorescence) filters. Species distribution in dual biofilms was assessed by pretreating the cell cultures with CellTrace Cell Proliferation Kits (Thermo Fisher Scientific, Europe) before inoculation to Ibidi slides. The bacterial cultures were stained with 1 µM CellTrace Far Red Dye (*A. naeslundii* CW) or 5 µM CellTrace CFSE Green Dye (*S. gordonii* CW) according to the manufacturer's protocol with support from a paper from Aherne et al. (34) with minor adaptations as follows. After the addition of CellTrace dye working solutions, cultures were incubated and protected from light for 1 h at 37°C, washed by centrifugation (12,000 rpm at RT, Eppendorf centrifuge 5,415 D), resuspended in sterile PBS, and inoculated as described under the biofilm culture section. CellTrace-labeled biofilms were then imaged using a Nikon Eclipse TE2000 spinning disc confocal microscope with a CFI Plan Apokromat 60× oil lens, numerical aperture 1.40 (Nikon, Japan), Prime 95B Scientific CMOS camera (Photometrics, Teledyne Photometrics, Tucson, AZ, USA), and SPECTRA X light engine (Lumencor Inc., OR, USA) for illumination at 60× magnification. Each of the biofilm triplicates was imaged at 10 different randomly selected positions each. The viability and species distribution was assessed by calculating the percentage green and red pixels in the images using the BioImage_L software (35). The biomass between replicate biofilms was monitored using a crystal violet biomass quantification assay for all replicates at the end of each experiment. After the collection of supernatants for nuclear magnetic resonance (NMR) analysis and sampling for culture to confirm survival of both species and exclude contamination, all wells were fixed by 30 min of incubation with 99% ethanol. The biofilms were then air-dried for approximately 10 min, rinsed three times gently with PBS, and stained for 5 min with 0.2% crystal violet (CV). Excess stain was removed by gentle rinsing with PBS. The stain that was retained in the biofilms was then dissolved in 33% acetic acid, and the absorbance was read at 570 nm.

### Nuclear magnetic resonance sample preparation, analysis, and metabolite annotation

Following incubation, the culture fluids were collected gently with a pipette, transferred to an Eppendorf tube and centrifuged at 5,000 rpm, 4°C for 5 min (Heraeus Fresco 17 centrifuge, Thermo Fisher Scientific, Europe) for removal of suspended cells. Supernatants were then replaced in fresh tubes and stored at −20°C until and during transport to the Swedish NMR Centre in Gothenburg for NMR analysis, where the frozen samples were thawed on ice. A total volume of 300 µl per sample was transferred manually to a deep well plate (Porvair catalog number 219,030), with wells containing 300 µl buffer {75 mM sodium phosphate, pH 7.4, 0.08% w/v TSP-d4 [3-(trimethylsilyl)propionic-2,2,3,3-d 4 acid sodium salt], 0.1% w/v sodium azide, 20% v/v deuterium oxide}. After sealing with a silicone lid, the samples were shaken for 5 min at 500 rpm, 12°C on a Thermomixer Comfort (Eppendorf, Nordic). To transfer 575 µl of each mixed sample to 5 mm SampleJet NMR tubes (Bruker Biospin, Ettlingen, Germany), a SamplePro Tube L (Bruker Biospin, Ettlingen, Germany) liquid handler was used. NMR data were acquired on a Bruker Avance NEO 600 MHz spectrometer equipped with a 5 mm QCI cryoprobe and a cooled SampleJet sample changer from five biologically independent replicates. During sample preparation, as well as in the spectrometer sample changer, sample racks were kept at 6°C up until measurement. To acquire 1D 1H data, the standard pulse sequence “zgespe” was used. The experiment encompassed a perfect echo sequence with excitation sculpting for water suppression. A total of 64 scans were collected into 64 k data points with a spectral width of 11,904 Hz, using an acquisition time of 2.692 s, a relaxation delay of 4 s, and eight dummy scans. The receiver gain was set to a fixed value of 101 and acquisition was performed at 25°C. The acquired data were zero-filled twice and an exponential line broadening of 0.3 Hz was added before Fourier transform and subsequent automated phasing and baseline correction. Spectra were referenced to the TSP-d4 signal at 0 ppm. TopSpin 4.1.4 (Bruker BioSpin, Ettlingen, Germany) was used to perform data acquisition and processing. ChenomX 9.0 (ChenomX Inc.) was used for metabolite signal annotation. The NMR data were imported into Matlab [MATLAB version: 9.13.0 (R2022b), MathWorks Inc., Natick, MA, USA] using the function rbnmr (36). The TSP-d4 peak was aligned between all spectra using the function ico_shift (37) and set at 0 ppm. The data were then imported to R using the package R.matlab. The readx1 package was used to import the corresponding metadata. The data were processed with the speaq package speaq (38). The getWaveletPeaks function with baselineThresh = 0, SNR.Th = 10, and include_nearbyPeaks = TRUE was used to select peaks. The PeakGrouper function with grouping.window.width = 200 and min.samp.grp = 10 was used for grouping. The SilhouetR function was run, and groups with a value < 0.5 were listed to be regrouped in a repeat run. The PeakFilling function was run filling in missing values with max.index.shift = 200. The output list was annotated and manually curated, leaving 55 peaks for analysis.

### Pathway enrichment analysis

Pathway enrichment analysis (PEA) was performed against the KEGG database (39) using MetaboAnalyst 6.0 (Montreal, QC, Canada) (40) against *Streptococcus pyogenes* M1 476 (serotype M1) reference metabolome (39), representing the closest related bacterial species to *A. naeslundii* and *S. gordonii* available. The identified compounds were tested against annotated cellular pathways in the reference metabolome to detect over-representations compared with what would be expected randomly based on the compound list.

### Statistical analyses

Species distribution was assessed by calculating the percentage of red (*A. naeslundii* CW) and green (*S. gordonii* CW) signals (CellTrace dye) in the SDCM images using the BioImage_L software (35) and reported as a mean % ± one standard deviation. The resulting values from the biomass CV assay are arbitrary and therefore only trends could be observed. Metabolites detected in the NMR analysis were determined to be present in a group if they were present in ≥2 of the five replicates. All statistical analyses were performed in R (41). Tests for normal distribution (Shapiro–Wilks) and equal distribution between groups (Levene's, visualization with histogram) of the metabolomic data (raw- and log-transformed) showed that the data were not normally distributed. Metabolite abundance was therefore compared using the nonparametric-related-sample Wilcoxon signed rank test for the comparison of two groups, or the related-sample Friedman's two-way analysis of variance by ranks with the Nemenyi post hoc test to correct for multiple tests when comparing three or more groups. The analyses were paired in order to compensate for batch effects between the different runs. Metabolite covariation was analyzed using a principal component analysis (PCA) using the “ggplot2” R package (42), with min-max-normalized confidence interval ellipses for each group. Explanatory factors were visualized with a Scree plot and a biplot. The PEA was based on node centrality network topology analysis, hypergeometric test, and over-representation analysis with a false discovery rate analysis to correct for multiple tests (analysis was performed using the software MetaboAnalyst 6.0).

## Results

This study investigates how the presence of the salivary mucin MUC5B affects the metabolic output during glucose metabolism in mono- and dual-species early biofilms of the two clinically isolated commensal bacteria, *A. naeslundii* CW and *S. gordonii* CW.

### Biofilms

#### Biofilm biomass, viability, and species distribution

**Figure 2:**
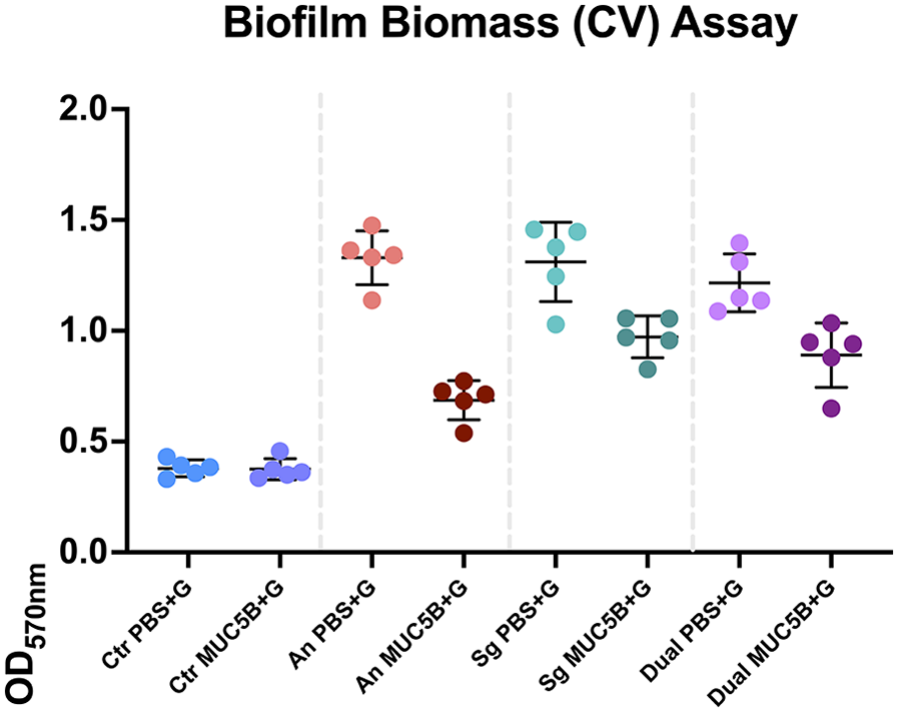
Absorbances read at 570 nm in the crystal violet (CV) biomass quantification assay of all replicates at the end of each experiment. The error bars show mean absorbance ± one standard deviation. Ctr, control; PBS + G, phosphate buffer with 20 mM glucose; MUC5B + G, 25% MUC5B in PBS with 20 mM glucose; An, *A. naeslundii* CW; Sg, *S. gordonii* CW; Dual, *A. naeslundii* CW and *S. gordonii* CW 1:1.

**Figure 3:**
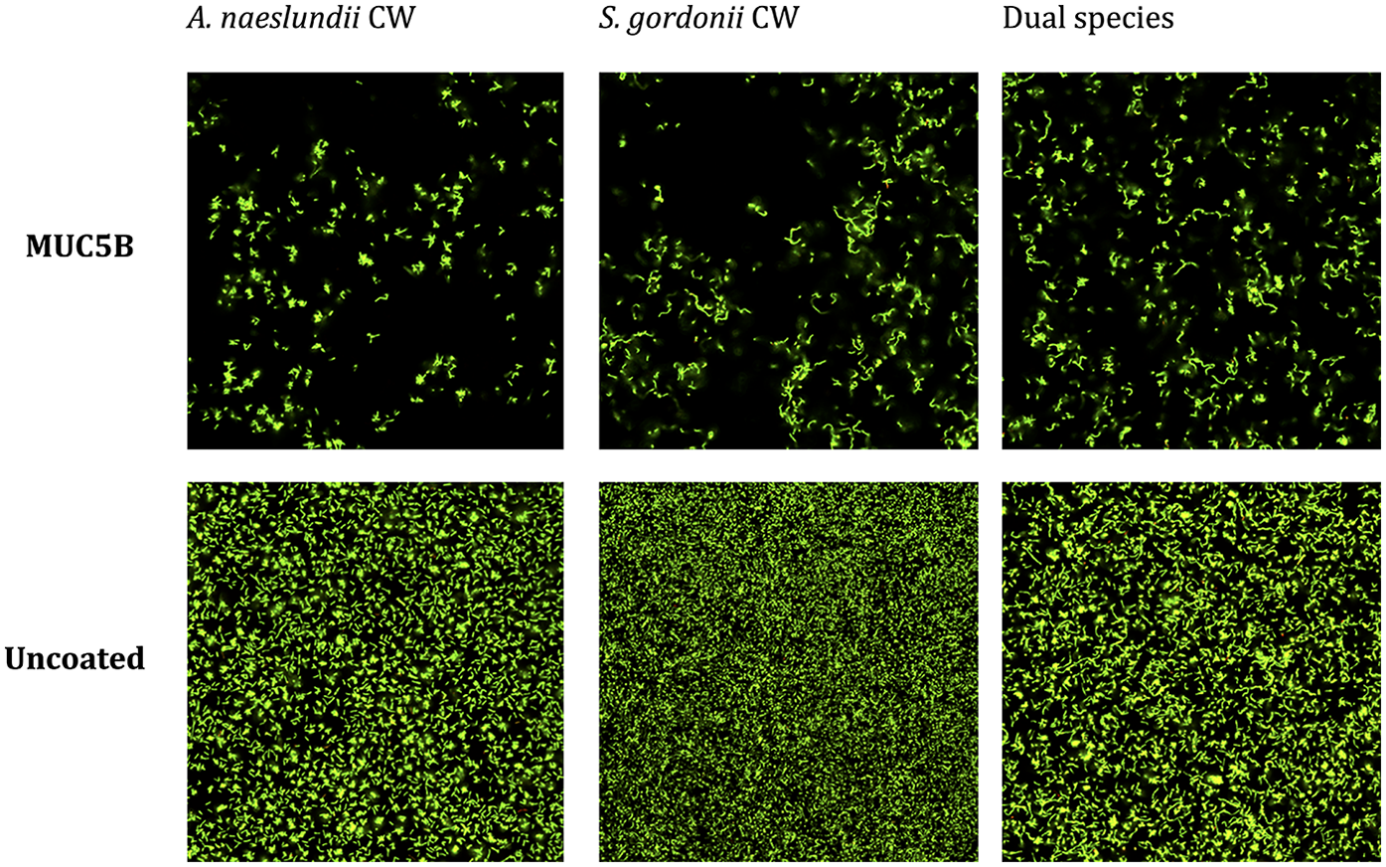
Viability of *A. naeslundii* CW and *S. gordonii* CW mono- and dual-species early biofilms on salivary MUC5B and uncoated surface after biofilm attachment phase, imaged using confocal scanning laser microscopy (BacLight LIVE/DEAD stain). Green = live cells. Red and yellow = dead or dying cells. Viability was >90% in all biofilms.

**Figure 4:**
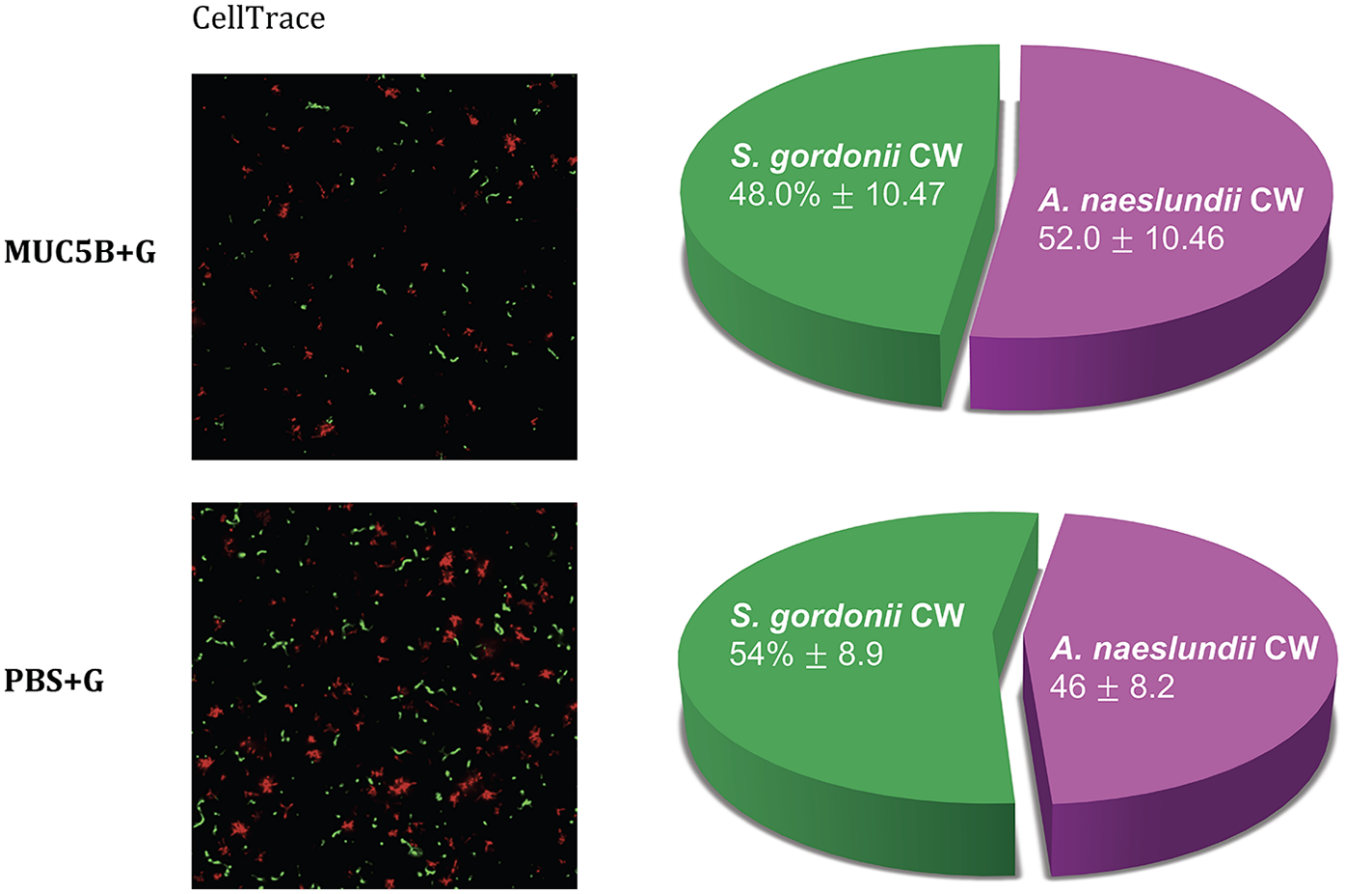
Species distribution in early dual-species biofilms in the presence and absence of MUC5B + G, imaged at the end of the experiments with CellTrace Far Red (red, *A. naeslundii* CW) and CFSE (green, *S. gordonii* CW) dye using spinning disc confocal microscopy. The pie charts show species distribution in the dual-species biofilms in the two different media, mean percentage ± one standard deviation. The differences were not significant.

The CV assay showed that the biomass of the replicate early biofilms was relatively consistent and that, as expected, biofilm formation on MUC5B (26) was generally lower than on uncoated hydrophilic plastic well surfaces (Figure 2). Confocal imaging showed that cell viability was >90% in all biofilms (Figure 3) and that the distribution of the two species was similar (Figure 4). Regrowth after reinoculation of samples from the wells on blood agar showed that all biofilms were viable at the end of the experiments, and that viable cells of both *A. naeslundii* and *S. gordonii* remained present in similar quantities in the dual-species biofilms.

#### Biofilm metabolomics

After the exclusion of duplets and artefacts, an analysis with NMR led to the detection of a total of 55 peaks that represented individual metabolites, of which 14 could be identified and annotated (NMR raw data have been uploaded after review for public access through the MetaboLights online repository, accession number MTBLS10078). In the *A. naeslundii* monospecies biofilms, the total number of metabolites detected was higher in the biofilms grown with MUC5B + G compared with PBS + G, while in the *S. gordonii* monospecies, as well as the dual-species biofilms, the number was lower (Table 1).

**Table 1:** Total number of metabolites detected under different conditions.

Species	Medium	Number of metabolites detected
*A. naeslundii* CW	PBS + G	33
*A. naeslundii* CW	MUC5B + G	35
*S. gordonii* CW	PBS + G	40
*S. gordonii* CW	MUC5B + G	37
Dual	PBS + G	34
Dual	MUC5B + G	28

PBS + G, phosphate buffer with 20 mM glucose; MUC5B + G, 25% MUC5B in PBS with 20 mM glucose; Dual, *A. naeslundii* CW and *S. gordonii* CW 1:1.

All annotated metabolites were end products of either in carbohydrate metabolism and pyruvate conversion (acetate, acetone, butyrate, ethanol, formate, lactate, propionate, pyruvate, and succinate) or in amino acid metabolism (2-oxoisocaproate, 2-oxoglutarate, alanine, and sarcosine).

#### Biofilm metabolomic profiles

**Figure 5:**
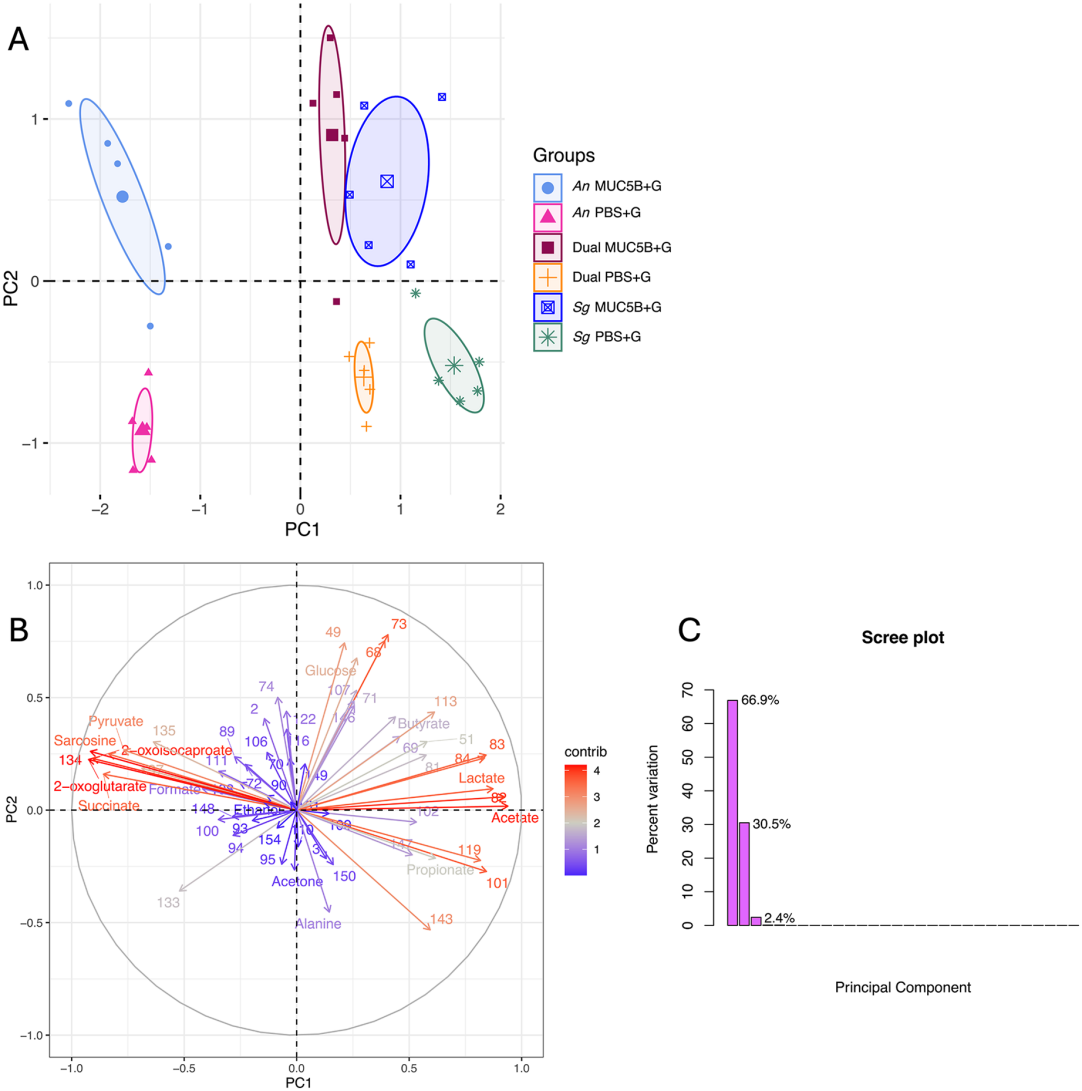
**(A)** Principal component analysis (PCA) of the first two components comparing the metabolomes of *A. naeslundii* CW and *S. gordonii* CW mono- and dual-species early biofilms grown in PBS + G and MUC5B + G media. The ellipses represent 95% confidence intervals around the center point after min-max normalization. **(B)** PCA biplot and **(C)** Screen plot of the PCA explanatory factors. R2X(1) = 66.9% and R2X(2) = 30.5% of variance explained. PBS + G, phosphate buffer with 20 mM glucose; MUC5B + G, 25% MUC5B in PBS with 20 mM glucose; An, *A. naeslundii* CW; Sg, *S. gordonii* CW; Dual, *A. naeslundii* CW and *S. gordonii* CW 1:1.

**Figure 6:**
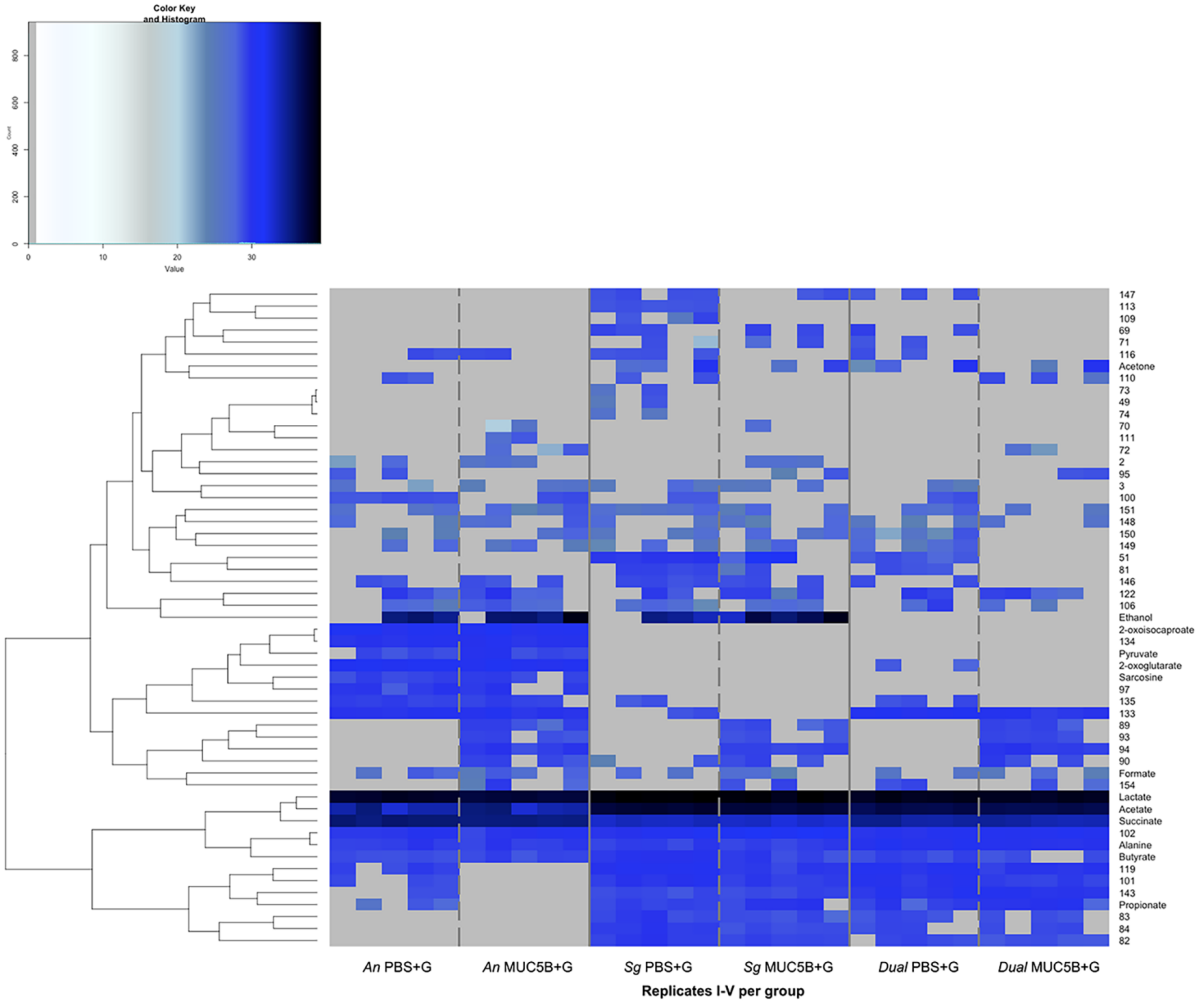
Metabolomes from *A. naeslundii* CW and *S. gordonii* CW mono- and dual-species early biofilms grown in the presence of PBS + G and MUC5B + G. Hierarchical clustering analysis of log2-normalized intensities for metabolites. Metabolites with signals that were detected in at least two of the five replicates within the groups are displayed. PBS + G, phosphate buffer with 20 mM glucose; MUC5B + G, 25% MUC5B in PBS with 20 mM glucose; An, *A. naeslundii* CW; Sg, *S. gordonii* CW; Dual, *A. naeslundii* CW and *S. gordonii* CW 1:1.

The PCA showed a clear separation between the overall metabolomes of all groups (Figure 5A), with the two principal components explaining 97.4% of variation (Figure 5C), and variation within groups was similar. Ten metabolites were common to all conditions (acetate, alanine, butyrate, formate, lactate, succinate, and unnanotated metabolites 102, 122, 148, and 151, Figure 6). Of these 10 core metabolites, even though the biofilm biomasses and surface coverages were similar within *A. naeslundii*, *S. gordonii*, and dual-species biofilms grown with PBS + G, and within biofilms grown with MUC5B + G (Figure 2), not unexpectedly, acetate was significantly more abundant in *S. gordonii* in PBS + G compared with *A. naeslundii* in PBS + G (*p* = 0.0028), and in *S. gordonii* in MUC5B + G compared with *A. naeslundii* in MUC5B + G (*p* = 0.028, Table 2). Butyrate was also significantly more abundant in *S. gordonii* in PBS + G compared with *A. naeslundii* in PBS + G (*p* = 0.047), and lactate was significantly more abundant in *S. gordonii* in MUC5B + G compared with *A. naeslundii* in MUC5B + G (*p* = 0.0.0028). Succinate was more abundant in *A. naeslundii* in PBS + G compared with *S. gordonii* in PBS + G (*p* = 0.009), but in *S. gordonii* in MUC5B + G compared with *A. naeslundii* in MUC5B + G (*p* = 0.009). Metabolite 102 was more abundant in *S. gordonii* in MUC5B + G compared with *A. naeslundii* in MUC5B + G (*p* = 0.0094). Additional statistically significant differences with low biological relevance are listed separately in the Supplementary Material (Supplementary Table S1).

**Table 2:** Comparison of metabolite abundance in different conditions.

Metabolite (peak nr)	Conditions with metabolite present						*p-*value	Significantly more abundant in
	An PBS + G	An MUC5B + G	Sg PBS + G	Sg MUC5B + G	Dual PBS + G	Dual MUC5B + G		
84			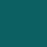	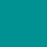	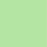	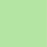	0.025**^b^** 0.036**^c^**	Sg PBS + G
Sarcosine (88)	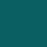	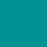					0.009**^a^**	An PBS + G
2-oxoglutarate (96)	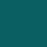	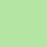			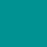		0.015**^b^** 0.012**^c^**	An PBS + G
97	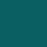	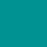					0.014**^a^**	An PBS + G
Succinate (98)	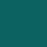 ^I^	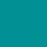 ^II^	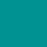 ^I^	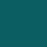 ^II^	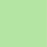	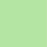	0.0002**^b^** 0.009**^c^**^I^ 0.009**^c^**^II^	An PBS + G^I^ Sg MUC5B + G^II^
Pyruvate (99)	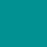	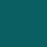					0.009**^a^**	An MUC5B + G
101	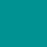 ^I,II^		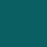 ^I^	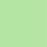	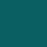 ^II^	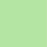	0.0023**^b^** 0.012**^c^**^I^ 0.003**^c^**^II^	Sg PBS + G^I^ Dual PBS + G^II^
102	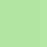	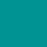	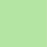	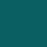	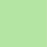	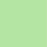	0.0013**^b^** 0.0094**^c^**	Sg MUC5B + G
Acetate (114)	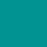 ^I^	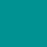 ^II^	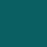 ^I^	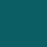 ^II^	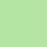	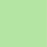	0.00031**^b^** 0.0028**^c^**^I^ 0.028**^c^**^II^	Sg PBS + G^I^ Sg MUC5B + G^II^
119	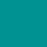 ^I,II^		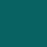 ^I^	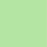	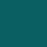 ^II^	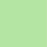	0.0037**^b^** 0.0061**^c^**^I^ 0.022**^c^**^II^	Sg PBS + G^I^ Dual PBS + G^II^
Lactate (123)	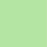	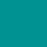	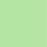	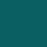	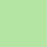	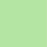	0.00047**^b^** 0.0028**^c^**	Sg MUC5B + G
Ethanol (131)	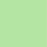	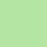	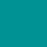	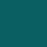			0.017**^b^** 0.025**^c^**	Sg MUC5B + G
133	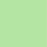	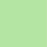	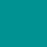		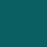	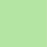	0.003**^b^** 0.0006**^c^**	Dual PBS + G
134	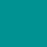	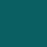					0.002**^a^**	An MUC5B + G
135	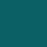	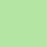	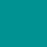		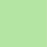		0.015**^b^** 0.025**^c^**	An PBS + G
143	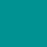		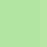	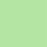	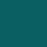	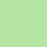	0.0019**^b^** 0.012**^c^**	Dual PBS + G
Butyrate (145)	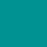	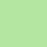	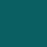	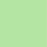	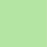	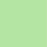	0.0079**^b^** 0.047**^c^**	Sg PBS + G

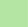
, present in condition; 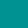, 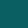, significant difference in abundance, higher abundance indicated by a darker shade. An, *Actinomyces naeslundii* CW; Sg, *Streptococcus gordonii* CW; dual, *Actinomyces naeslundii* CW, and *Streptococcus gordonii* CW together; MUC5B + G, Biofilm was grown on a salivary mucin MUC5B-coated surface with MUC5B + G medium; PBS + G = Biofilm was grown on an uncoated surface with PBS + G medium, ^I/II^ = Conditions that displayed significant differences in multiple comparisons.

^a^ *p*-Value from the Wilcoxon test for two related samples.

^b^ *p*-Value from related-samples Friedman's analysis of variance by ranks.

^c^ *p*-Value from Nemenyi post hoc test after significant outcome from related-samples Friedman's analysis of variance by ranks. NMR peak number is stated as the metabolite name for unannotated metabolites or in parenthesis for annotated metabolites.

In addition to the core metabolome, a number of metabolites were present only in certain conditions (Figure 6). Of these, an additional 12 metabolites showed significant differences in abundance between conditions when comparing between biofilms (*A. naeslundii*, *S. gordonii*, and dual species) in the same medium (PBS + G or MUC5B) or within biofilms when comparing between media (Table 2). Metabolite 84 was present only in the *S. gordonii* monospecies and the dual-species biofilms, with a significantly higher abundance in *S. gordonii* in PBS + G compared with *S. gordonii* in MUC5B + G (*p* = 0.036). Sarcosine and metabolite 97 were present only in the *A. naeslundii* monospecies biofilms with a significantly higher abundance in PBS + G compared with MUC5B + G (*p* = 0.009 and 0.014). Pyruvate and metabolite 134 were present only in the *A. naeslundii* monospecies biofilms with a significantly higher abundance in MUC5B + G compared with PBS + G (*p* = 0.009 and 0.002). 2-Oxoglutarate was present in both *A. naeslundii* monospecies biofilms (PBS + G and MUC5B + G) and the dual-species biofilm (PBS + G), with significantly higher abundance in *A. naeslundii* in PBS + G compared with the dual species (*p* = 0.012). Metabolites 101 and 119 were present in all conditions, except for *A. naeslundii* MUC5B + G, with significantly higher abundance in *S. gordonii* PBS + G compared with *A. naeslundii* PBS + G (*p* = 0.012 and 0.0061), and in dual-species PBS + G compared with *A. naeslundii* PBS + G (*p* = 0.003 and 0.022). Ethanol was present only in the monospecies biofilms of the two species in PBS + G and MUC5B + G, but missing in the dual-species biofilms in both PBS + G and MUC5B + G (*p* = 0.025). Metabolite 133 was present in all conditions, except for *S. gordonii* in MUC5B + G, with significantly higher abundance in dual-species biofilms in PBS + G compared with *S. gordonii* in PBS + G (*p* = 0.0006). Metabolite 135 was present in all conditions, except for *S. gordonii* in MUC5B + G and dual-species biofilm in MUC5B + G, with a significantly higher abundance in *A. naeslundii* in PBS + G compared with *S. gordonii* in PBS + G (*p* = 0.025). Finally, Metabolite 143 was present in all conditions except for *A. naeslundii* MUC5B + G, with a significantly higher abundance in dual species in PBS + G compared with *A. naeslundii* PBS + G 0.012). Alanine, formate, and unnanotated metabolites, 122, 148, and 151 were produced in all conditions with no significant differences in abundance.

### Metabolite covariation

The PCA showed a number of metabolites that covaried to distinguish the mono and dual *A. naeslundii* and *S. gordonii* biofilms grown with PBS + G and MUC5B + G (Figures 5A,B). The metabolites that seemed to distinguish the biofilms grown with MUC5B + G from the biofilms grown with PBS + G, in general, were mainly unannotated metabolites 68 and 73 (Figure 5B). The metabolomes for the dual-species biofilms were overall more similar to the *S. gordonii* metabolomes, while the *A. naeslundii* metabolomes were more different. The metabolites that seemed to distinguish the *A. naeslundii* biofilms from the *S. gordonii* and dual-species biofilms were acetate, lactate, pyruvate, succinate (all involved in pyruvate metabolism) and sarcosine, 2-oxoisocaproate, and 2-oxoglutarate (all involved in amino acid metabolism). Of the organic acids, lactate and acetate were abundant in *S. gordonii* and dual-species biofilms, while succinate was abundant in *A. naeslundii*.

### Pathway enrichment analysis

**Figure 7:**
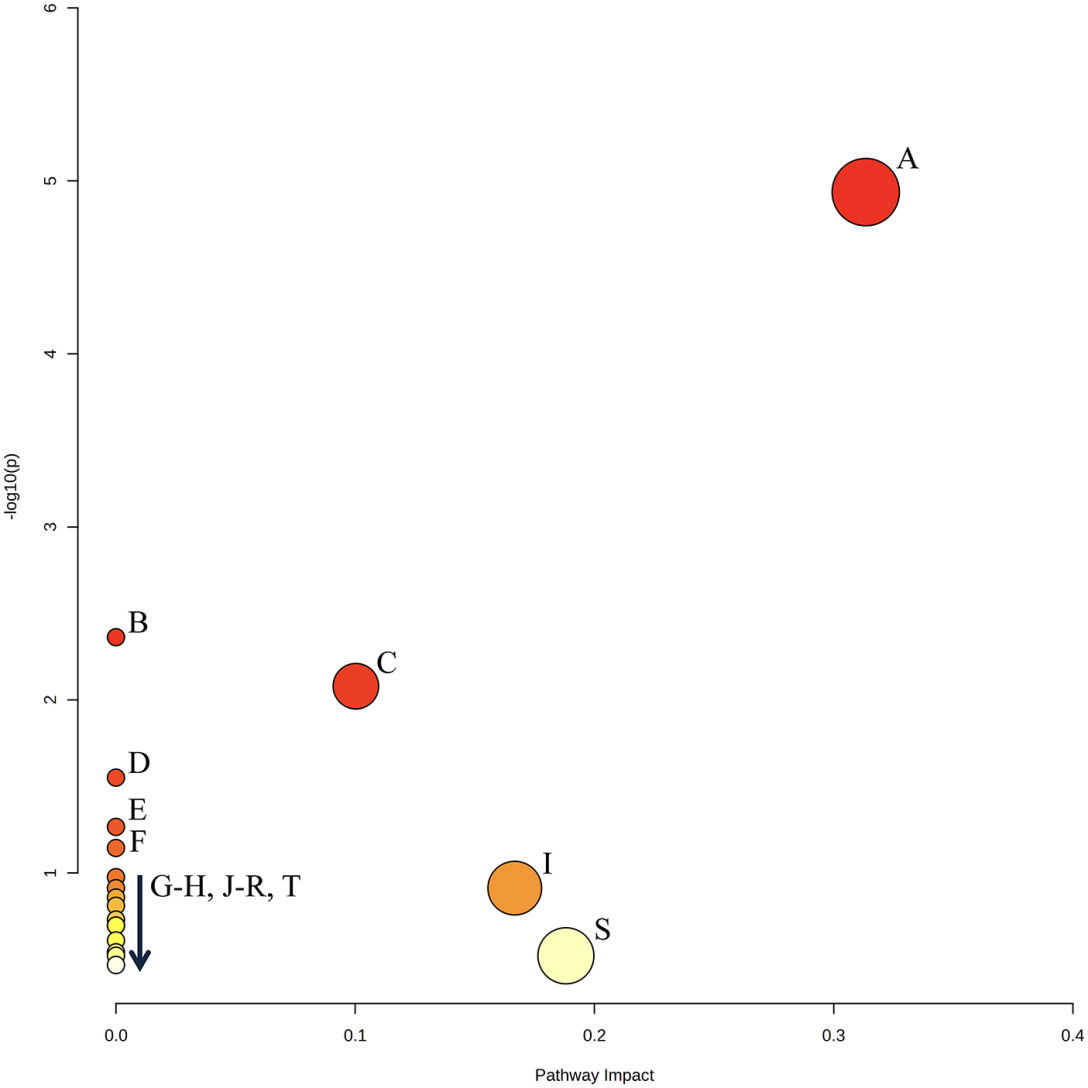
Graphical output of the top 20 enriched pathways identified by PEA (see Table 3 for annotations). The *Y* axis displays transformed *p*-values for over-representation analysis [−log10(p)], and the *X* axis displays the pathway impact score relative to the global reference metabolomic network, calculated from the node centrality network topology analysis. Pathway annotation is listed in Table 3. Created with MetaboAnalyst 6.0.

Pathway enrichment analysis of the metabolites that were differently expressed in early *A. naeslundii*, *S. gordonii*, and dual-species biofilms grown with PBS + G or MUC5B + G showed that pyruvate metabolism was significantly over-represented (Figure 7, Table 3, FDR = 0.00075). This pathway also had the highest impact score (0.31). The five metabolites that were matched to the pyruvate metabolism pathway were acetate, butyrate, formate, lactate, and pyruvate. The remaining metabolic pathways listed in Table 3 were also matched to regulated metabolites but showed no statistically significant over-representation. Glycolysis/gluconeogenesis (C), sulfur metabolism (I), and starch and sucrose metabolism (S) also showed results in the impact scores (impact scores of 0.1, 0.17, and 0.19 respectively).

**Table 3:** List of the top 20 enriched pathways identified by PEA as shown in Figure 7, ranked by significance.

Pathway name	Match status	*p* (raw)	−log10(p)	FDR	Impact	Metabolites
A. Pyruvate metabolism	5/21	1.1605E-5	4.9354	7.5433E-4	0.3134	Acetate, ethanol, formate, lactate, and pyruvate
B. Butanoate metabolism	3/20	0.0043388	2.3626	0.14101	0.0	Butyrate, pyruvate, succinate
C. Glycolysis/gluconeogenesis	2/25	0.0083309	2.0793	0.1805	0.10027	Pyruvate, ethanol
D. Alanine, aspartate, and glutamate metabolism	2/15	0.02813	1.5508	0.45711	0.0	Alanine, pyruvate
E. Taurine and hypotaurine metabolism	1/3	0.054238	1.2657	0.70509	0.0	Acetate
F. Tyrosine metabolism	1/4	0.071719	1.1444	0.77696	0.0	Succinate
G. Valine, leucine, and isoleucine biosynthesis	1/6	0.10581	0.97546	0.87291	0.0	2-Oxoisocaproate
H. Citrate cycle (TCA cycle)	1/7	0.12243	0.9121	0.87291	0.0	Pyruvate
I. Sulfur metabolism	1/7	0.12243	0.9121	0.87291	0.16667	Acetate
J. Arginine biosynthesis	1/8	0.13878	0.85769	0.87291	0.0	2-Oxoglutarate
K. Streptomycin biosynthesis	1/9	0.15484	0.81011	0.87291	0.0	Pyruvate
L. Valine, leucine, and isoleucine degradation	1/11	0.18617	0.73009	0.87291	0.0	2-Oxoisocaproate
M. Glycine, serine, and threonine metabolism	1/12	0.20144	0.69585	0.87291	0.0	Sarcosine
N. Glyoxylate and dicarboxylate metabolism	1/12	0.20144	0.69585	0.87291	0.0	Acetate
O. Nicotinate and nicotinamide metabolism	1/12	0.20144	0.69585	0.87291	0.0	Succinate
P. Lipoic acid metabolism	1/15	0.24571	0.60957	0.99821	0.0	Pyruvate
Q. Pentose phosphate pathway	1/18	0.28776	0.54096	1.0	0.0	Pyruvate
R. Methane metabolism	1/19	0.3013	0.521	1.0	0.0	Acetate
S. Starch and sucrose metabolism	1/19	0.3013	0.521	1.0	0.18806	Pyruvate
T. Cysteine and methionine metabolism	1/22	0.34055	0.46782	1.0	0.0	Pyruvate

Match status indicates the number of metabolites from experimental data matched to the pathway/total number of compounds in the pathway; *p*-values [raw, −log10(p), FDR] represent significance from over-representation analysis; impact indicates the  pathway impact score relative to the global reference metabolomic network, calculated bioinformatically from node centrality network topology analysis; metabolites indicates the name of metabolites that were matched to the pathway. Created with MetaboAnalyst 6.0.

## Discussion

To investigate how early mono- and dual-species biofilms of oral *A. naeslundii* and *S. gordonii* clinical isolates metabolized glucose in the presence of the complex salivary mucin MUC5B, this study employed NMR-based metabolomics with the interpretation of network integration. Untargeted NMR data analysis for metabolite detection and annotation and *in silico* studies of biochemical relationships between metabolites and cellular processes was performed to study functional differences in the early biofilm metabolomes. Studying the metabolic interactions between commensal members of oral biofilms and modulatory effects of host factors such as glycoproteins in saliva during the metabolism of substrates that are potential drivers of dysbiosis, such as glucose, is essential to understand the roles of oral microbial ecosystems in oral health and disease.

The consistency between the biomasses of the replicate biofilms (Figure 2), cell viability (Figure 3), regrowth of sampled biofilms on blood agar, and the maintained equal species distribution in dual-species biofilms (Figure 4) showed that the model was robust. Previous work has shown that the presence of MUC5B on the surfaces and in the medium reduces biofilm adhesion compared with uncoated well surfaces (26) that are similar to tooth enamel, and dentine hydroxyapatite possesses hydrophilic properties, and here, this pattern was found to also persist in the presence of glucose (Figure 2). Since MUC5B reduced biofilm biomass while supporting continued colonization by both commensals, even when growing the bacteria with glucose that represents a nutrient source that supports saccharolytic metabolism and EPS production, which would otherwise be expected to increase the volume of the biofilms (17), it can be hypothesized that salivary MUC5B acts as a part of innate immune mechanisms to promote oral biofilm eubiosis by maintaining biodiversity through a coadhesion of commensals, while regulating the total amount of biofilm biomass. Evidence of this mechanism for host protection has also been reported and discussed in other studies (25, 27, 43).

In the current study, the presence of MUC5B during glucose metabolism elicited differences in the early biofilm metabolomic profiles compared with PBS (Figures 5, 6). All annotated metabolites from the NMR analysis were intermediates either in carbohydrate metabolism and pyruvate conversion (acetate, acetone, butyrate, ethanol, formate, lactate, propionate, pyruvate, and succinate) or in amino acid metabolism (2-oxoisocaproate, 2-oxoglutarate, alanine, and sarcosine). The identification of metabolites related to these pathways is common in metabolomic studies of oral bacteria (44), and is not unexpected in biofilm metabolomes from the two saccharolytic species *A. naeslundii* and *S. gordonii*, especially in the presence of glucose, which is one of the primary substrates for carbohydrate metabolism in these species and considering that pyruvate conversion represents a junction point between glycolysis and amino acid metabolic chains. There was no significant difference in glucose abundance between mono- and dual-species biofilms of the two bacterial species at the end of the experiments, indicating similar levels of glucose consumption. The 42 metabolites from the NMR analysis that could not be annotated, mainly due to the peaks being too weak for identification with 2D NMR techniques, are also likely a part of additional cellular pathways that could not be identified at this time.

Ten metabolites were common to all conditions (acetate, alanine, butyrate, formate, lactate, succinate, and unnanotated metabolites 102, 122, 148, and 151, Figure 6), and the presence of these 10 metabolites in all biofilms suggests that they represent a core metabolome shared by *A. naeslundii* and *S. gordonii* with the continued presence in dual-species biofilms. It is somewhat surprising that succinate was also present in the *S. gordonii* monospecies biofilms, since it is a metabolite that has traditionally been used to distinguish *Actinomyces* species from oral streptococci in phenotypic tests. The enzyme used by *Actinomyces* to produce succinate, succinate dehydrogenase, was not found to be annotated for any *S. gordonii* species in the protein sequence database UniProt (45); however, for the type strain *S. gordonii* Challis CH1, the gene that codes for this enzyme had been annotated in the KEGG genome (39). In a previous whole-genome sequencing of the *S. gordonii* CW clinical isolate (Genbank accession number CP113953), the genes coding for four enzymes involved in alternative succinate production from nucleotide biosynthesis, dihydroorotate dehydrogenase, argininosuccinate synthase, argininosuccinate lyase, and adenylosuccinate synthase, were annotated. These enzymes were also annotated for the type strain *S. gordonii* CH1 Challis in UniProt. Hence, even though the precise mechanisms for succinate production in these biofilms are presently unknown, the detection of succinate also in *S. gordonii* CW monospecies biofilms with 20 mM glucose with and without MUC5B, although initially surprising, can be considered reliable. In our previous study (26), succinate was not present in the *S. gordonii* CW monospecies biofilms on MUC5B in the absence of glucose, indicating that free glucose is probably necessary to induce succinate production. Future studies are needed to bring the details of these mechanisms into light. These findings underline the importance of combining several parameters for species identification by phenotypic tests, such as morphology (shown for *S. gordonii* CW and *A. naeslundii* CW in Figure 1) and a good array of metabolic reactions, as well as the benefits of also utilizing genomics, such as 16S rRNA and/ or whole-genome sequencing as previously performed for the strains employed in this study (Genbank accession numbers OQ625895 and CP113953 for *S. gordonii* CW and OQ625896 and CP113787 for *A. naeslundii* CW), to identify species from clinical samples with high precision.

In addition to the shared metabolites, condition-specific regulations to the metabolite abundances were also detected. Of these 10 core metabolites, even though the biofilm biomasses were similar within *A. naeslundii, S. gordonii,* and dual-species biofilms grown with PBS + G, and within biofilms grown with MUC5B + G (Figure 2), acetate and butyrate were significantly more abundant in *S. gordonii* in PBS + G compared with *A. naeslundii* in PBS + G (*p* = 0.0028 and 0.047, Table 2). Acetate, lactate, and metabolite 102 were also more abundant in *S. gordonii* in MUC5B + G compared with *A. naeslundii* in MUC5B + G (*p* = 0.028, 0.0.0028, and 0.0094). Succinate was more abundant in *A. naeslundii* in PBS + G compared with *S. gordonii* in PBS + G (*p* = 0.009), but interestingly in *S. gordonii* in MUC5B + G compared with *A. naeslundii* in MUC5B + G (*p* = 0.009). Hence, significant differences in metabolite abundance among metabolites present in all conditions was detected only between the two bacterial species. Of the metabolites that were not present in all conditions, on the other hand, significant differences in abundance elicited by the presence of MUC5B was detected. In the conditions where they were each present (Table 2), pyruvate, ethanol, and metabolite 134 were present in significantly higher abundance in the presence of MUC5B + G (*p* = 0.009, 0.025, and 0.002), while metabolites 84, 97, and sarcosine were present at significantly lower abundance (*p* = 0.036, 0.014, and 0.009).

There were also some metabolites that were present or missing only in biofilms grown with MUC5B + G compared with PBS + G (Figure 6). Of these, one was unique to the biofilms grown in MUC5B + G (metabolite 72) and eight were unique to biofilms grown in PBS + G (metabolites 49, 73, 74, 89, 93, 94, 109, 113). Alanine, formate, and unnanotated metabolites, 122, 148, and 151 were produced in all conditions with no significant differences in abundance. This suggests a straightforward accumulative effect from the production of each individual species in the dual-species biofilm. These metabolites represent a shared core metabolomic profile unaffected by *A. naeslundii* and *S. gordonii* co-adhesion. Studies often report differences in metabolomes between bacterial populations (46, 47), but a mapping of shared core metabolomes is also important to facilitate the identification of deviations.

**Figure 8:**
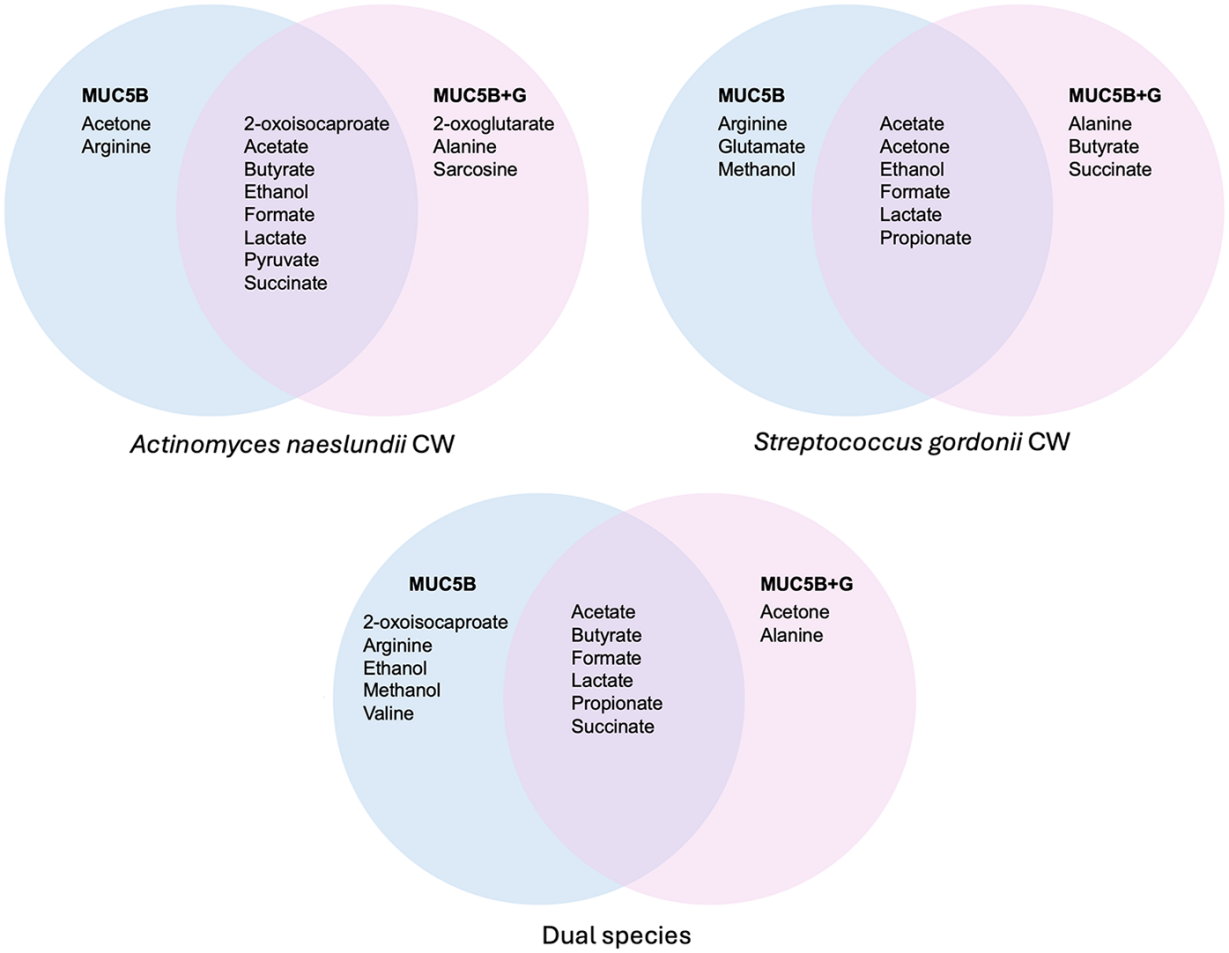
Comparison of the annotated metabolites in the metabolomes of *A. naeslundii* CW, *S. gordonii* CW, and dual-species biofilms grown on MUC5B-coated surfaces with 25% MUC5B medium with or without 20 mM glucose.

In a previous publication from our laboratory using similar methodology (26), a total of 27 individual NMR peaks were detected in *A. naeslundii* CW, *S. gordoniiI CW*, and dual-species early biofilms grown on MUC5B-coated surfaces with 25% salivary MUC5B-medium without the addition of glucose, of which 14 were identified and annotated. Of the metabolites annotated in these MUC5B biofilms with or without added glucose, the majority (10) of the identified metabolites were shared, but the addition of 20 mM glucose to MUC5B media also elicited some unique metabolites (Figure 8).

When grown with MUC5B without glucose, the amino acid valine was released from the MUC5B protein core, but only in dual biofilms with both *A. naeslundii* and *S. gordonii* CW together, as a consequence of synergistic metabolism (26). In the presence of glucose, however, valine could no longer be detected in mono- or dual-species biofilms of *A. naeslundii* and *S. gordonii* CW (Figure 6), and no other metabolite was unique to the dual-species biofilms. Another amino acid however, alanine, was present in all mono- and dual-species biofilms of *A. naeslundii* and *S. gordonii* CW, but only in the presence of glucose (Figures 6, 8). This supports the concept of advanced complexity in metabolic coordination that is being highlighted in recent studies (48) and exemplifies that there is a regulatory interplay both between interacting bacterial species and as a consequence of responses to nutritional substrates in the environment.

All groups were clearly separated on the PCA, and variation within groups was similar (Figure 5A). The PCA also revealed a number of metabolites that covaried to distinguish the mono- and dual *A. naeslundii* and *S. gordonii* biofilms grown with PBS + G and MUC5B + G (Figure 5B). The main metabolites that distinguished the biofilms grown with MUC5B + G from the biofilms grown with PBS + G, in general, were the unannotated metabolites 68 and 73. The metabolomes for the dual-species biofilms were more similar overall to the *S. gordonii* metabolomes, while the *A. naeslundii* metabolomes were more different. The metabolites that distinguished the *A. naeslundii* biofilms from the *S. gordonii* and dual-species biofilms were all involved in pyruvate metabolism (acetate, lactate, pyruvate, succinate) or amino acid metabolism (sarcosine, 2-oxoisocaproate, and 2-oxoglutarate). Of the organic acids, lactate and acetate were abundant in *S. gordonii* and dual-species biofilms, while succinate was abundant in *A. naeslundii*, which is to be expected, since *S. gordonii* is a known producer of acetate and lactate, especially in the presence of glucose (16), and succinate is one of the characteristic metabolic end products used in routine phenotypic identification of *Actinomyces* species from clinical samples. The PCA thus allowed for the identification of key metabolites that contributed most to the separation of the biofilm groups, thereby providing insights into specific metabolic pathways influenced by MUC5B. PCA was employed to analyze the metabolomic data due to its ability to reduce the dimensionality of complex datasets, while retaining most of the variations present in the data. The principal components highlight the most significant patterns in the data, which facilitates the identification of distinct metabolic profiles and underlying trends that might not be immediately apparent. The use of PCA in this context not only ensured a robust and interpretable analysis but also facilitated a more comprehensive understanding of the biochemical interactions within the biofilms.

The PEA of the metabolites that were differently expressed in early *A. naeslundii*, *S. gordonii*, and dual-species biofilms grown with PBS + G or MUC5B + G showed that pyruvate metabolism was the pathway that was significantly over-represented (Figure 7, Table 3, FDR = 0.00075) and had the highest impact score (0.31). This was not unexpected from biofilm formation of two mainly saccharolytic species *A. naeslundii,* and *S. gordonii* (8, 14) in the presence of glucose. The metabolomes of biofilms grown with MUC5B with (in this study) or without glucose [in our previous study (26)] were mostly shared, but displayed some differences in which metabolites were present (Figure 8) (26). Interestingly, the metabolites that were unique when comparing the biofilms grown in MUC5B with or without glucose were mainly amino acids or amino acid degradation products and not derived from glucose (Figure 8). This indicates that the metabolic regulations caused by the presence of MUC5B during glucose metabolism are intricate and affect numerous cellular pathways, not only pyruvate conversion as suggested by the PEA. MUC5B has previously been shown to promote proteolytic activity in oral bacteria (32, 33, 49). The production of alkali end products from maintained proteolytic activity supported by MUC5B would help counteract the cariogenic pH drops caused by glucose metabolism and thereby protect against cariogenic dysbiosis.

The metabolites from the biofilms that were formed with glucose with or without MUC5B in the current study were also mainly shared (Figure 6). None of the metabolites that were present only in one of the conditions were annotated (MUC5B + G metabolite 72, PBS + G metabolites 49, 73, 74, 89, 93, 94, 109, and 113) and therefore did not give any additional information about what cellular pathways may be regulated. Forty-two of the metabolites from the NMR analysis in the current study and 13 in our previous study (26) could not be annotated, and could therefore not be used in the over-representation analyses. Therefore there are likely additional pathways of relevance to the regulatory mechanisms of MUC5B on microbial metabolism that remain to be identified in future studies. The annotation of these metabolites was not possible due to weak signal and limited coverage in the spectral database ChenomX-HMDB used for annotation, a common issue in studies of bacterial metabolomes where unnamed metabolites are often reported (44). Future developments in experimental techniques and curated databases will facilitate better identifications.

In a previous study from our laboratory (26), *A. naeslundii* CW and *S. gordonii* CW seemed to cooperate by taking advantage of each other's enzymatic complementarity in their metabolism, a type of interplay that has been suggested to promote eubiotic diversity in mixed oral biofilms (30, 50). In the current study, the abundance of a number of metabolites were seemingly regulated by synergistic metabolic interaction between *A. naeslundii* and *S. gordonii* in the dual-species conditions. 2-Oxoglutarate was present in both *A. naeslundii* monospecies biofilms (PBS + G and MUC5B + G) and the dual-species biofilm in PBS + G, with significantly higher abundance in *A. naeslundii* in PBS + G compared with the dual species (*p* = 0.012). Metabolites 101 and 119 were present in all conditions, except for *A. naeslundii* MUC5B + G, with significantly higher abundance in dual-species PBS + G compared with *A. naeslundii* PBS + G (*p* = 0.003 and 0.022). Ethanol was present only in the monospecies biofilms of the two species both in PBS + G and in MUC5B + G, but missing in the dual-species biofilms in both PBS + G and MUC5B + G (*p* = 0.025). Metabolite 133 was present in all conditions, except for *S. gordonii* in MUC5B + G, with significantly higher abundance in dual-species biofilms in PBS + G compared with *S. gordonii* in PBS + G (*p* = 0.0006). Metabolite 143 was present in all conditions, except for *A. naeslundii* MUC5B + G, with significantly higher abundance in dual species in PBS + G compared with *A. naeslundii* PBS + G (*p* = 0.012). Considering that the biomasses were similar within corresponding biofilms (Figure 2), and the proportion of each species in the dual-species biofilms was approximately the same, suggests that when *A. naeslundii* and *S. gordonii* formed biofilms together, metabolic interactions beyond what would be expected from the sum of the two monospecies occurred. Despite the large metabolic resemblance between these two saccharolytic genera, previous studies have shown that their species and strains also differ in some respects, such as in a number of enzymes for transport and metabolism of carbohydrates (14, 19). Hence, as supported by the results in this and our previous paper (26), when members of oral biofilms such as *Actinomyces* and *Streptococcus* species form biofilms together, these organisms can cooperate to perform a synergistic metabolism of nutrients. In the mouth, such interactions, one example being the release of pyruvate leading to a detoxification of H_2_O_2_ (51), may increase their competitiveness in dental plaque (11, 52).

The biofilm model employed in this work enabled the study of microbial interactions and adaptations during early biofilm establishment in the presence of the salivary mucin MUC5B, with the ambition to increase the resemblance to *in vivo* conditions, when compared with traditional planktonic models with cultures in simplified media without the presence of components from human saliva. To better understand oral biofilm physiology and maturation, the study of host-mediated regulation of oral microbial biofilm activity, such as effects on metabolomes during glucose metabolism in the presence of salivary mucin MUC5B, is of great importance. Metabolomic responses in oral commensals to salivary MUC5B have been found to help regulate oral host-biofilm homeostasis, e.g., through interactions with mucin glycans (53). More studies are needed to understand these relationships during early biofilm formation and establishment, as well as during longer time periods to monitor effects on biofilm maturation and succession and assess their roles in health and disease. As also suggested in previous publications (13, 18, 40), further methodological developments are needed to optimize gene, protein, and metabolite identification and annotation in oral bacteria. Studies in these areas would contribute to the development of new and improved treatment and prevention strategies against oral biofilm–induced diseases.

## Conclusions

Salivary mucin MUC5B seems to act as part of innate immune mechanisms to promote oral biofilm eubiosis by maintaining biodiversity through a coadhesion of commensals, while regulating the total amount of biofilm biomass. Alanine, formate, and unnanotated metabolites, 122, 148, and 151, were produced in all conditions with no significant differences in abundance and thereby represent a shared core metabolomic profile unaffected by the presence or absence of MUC5B or by *A. naeslundii* and *S. gordonii* coadhesion. Significant differences in metabolite abundance elicited by the presence of MUC5B were detected. The main metabolites that distinguished the biofilms grown with MUC5B + G were the unannotated metabolites 68 and 73. In the conditions where they were each present, pyruvate, ethanol, and metabolite 134 were significantly more abundant in the presence of MUC5B + G, while metabolites 84, 97, and sarcosine were significantly less abundant. The PEA of the metabolites that were differently expressed in early *A. naeslundii*, *S. gordonii*, and dual-species biofilms grown with PBS + G or MUC5B + G showed that pyruvate metabolism was the pathway that was significantly over-represented. Because of the large proportion of metabolites that could not be annotated, there are likely additional pathways of relevance to the regulatory mechanisms of MUC5B on microbial metabolism that remain to be identified in future studies. Studying the metabolic interactions between commensal members of oral biofilms and modulatory effects of host factors such as glycoproteins in saliva during metabolism of substrates that are potential drivers of dysbiosis, such as glucose, is essential to understand the roles of oral microbial ecosystems in oral health and disease.

## Data Availability

The datasets presented in this study can be found in online repositories. The names of the repository/repositories and accession number(s) can be found in the article/Supplementary Material.
