# Supplementary material for: MUC5B modulation of early oral biofilm glucose metabolism
Source: Front Oral Health. 2025 Feb 11;6:1516025. doi: 10.3389/froh.2025.1516025 (PMC11850524; doi:10.3389/froh.2025.1516025)
Supplement: Supplementary file 1 [file Datasheet1.pdf]

Supplemental material

Statistically significant findings with low biological significance

Table S1. Comparison of metabolite abundance in different conditions with no significant findings or with no findings of biological meaning. An= *Actinomyces naeslundii* CW, Sg= *Streptococcus gordonii* CW, dual = *Actinomyces naeslundii* CW and *Streptococcus gordonii* CW together, MUC5B+G= Biofilm was grown on salivary mucin MUC5B-coated surface with MUC5B+G medium, PBS+G= Biofilm was grown on uncoated surface with PBS+G medium, [I/II]= Conditions that displayed significant differences in multiple comparisons, a= P value from Wilcoxon test for two related samples, b= P value from related-samples Friedman's analysis of variance by ranks, c= P value from Nemenyi posthoc test after significant outcome from related-samples Friedman's analysis of variance by ranks.

Metabolite (peak nr)	Conditions with metabolite present	P value	Condition where metabolite was more abundant
**Formate (1)**	*All*	0.52	-
**2**	An PBS+G, An MUC5B+G, Sg PBS+G, Dual PBS+G, Dual MUC5B+G	0.93	-
**3**	An PBS+G, An MUC5B+G, Sg PBS+G, Sg MUC5B+G, Dual PBS+G	0.50	-
**51**	Sg PBS+G, Sg MUC5B+G, Dual PBS+G	0.25	-
**69**	Sg PBS+G, Sg MUC5B+G, Dual PBS+G	1	-
**71**	Sg PBS+G, Sg MUC5B+G, Dual PBS+G	0.93	-
**72**	An MUC5B+G, Dual MUC5B+G	0.06[a]	-
**81**	Sg PBS+G, Sg MUC5B+G, Dual PBS+G	0.128	-
**82**	Sg PBS+G[I], Sg MUC5B+G, Dual PBS+G, Dual MUC5B+G[I]	0.005[b], 0.0034[c]	Sg PBS+G
**83**	Sg PBS+G[I], Sg MUC5B+G, Dual PBS+G, Dual MUC5B+G[I]	0.041[b], 0.050[c]	Sg PBS+G
**89**	An MUC5B+G, Sg MUC5B+G, Dual MUC5B+G	0.45	-

**90**	An MUC5B+G, Sg PBS+G, Sg MUC5B+G, Dual MUC5B+G	0.42	-
**93**	An MUC5B+G, Sg MUC5B+G, Dual MUC5B+G	0.37	-
**94**	An MUC5B+G, Sg MUC5B+G, Dual MUC5B+G	0.17	-
**95**	An PBS+G, Sg MUC5B+G, Dual MUC5B+G	0.81	-
**Succinate (98)**	*All:* An PBS+G[I], An MUC5B+G[II], Sg PBS+G[II], Sg MUC5B+G[I], Dual PBS+G, Dual MUC5B+G	0.0002[b], 0.001[cI], 0.047[cII]	Sg MUC5B+G[I], An MUC5B+G[II]
**100**	An PBS+G, An MUC5B+G, Sg PBS+G, Dual PBS+G	0.09	-
**102**	*All:* An PBS+G[I], An MUC5B+G, Sg PBS+G, Sg MUC5B+G[I], Dual PBS+G, Dual MUC5B+G	0.0013[b], 0.0014[c]	Sg MUC5B+G
**Acetone (103)**	Sg PBS+G, Sg MUC5B+G, Dual PBS+G, Dual MUC5B+G	0.224	-
**106**	*All*	0.39	-
**110**	An PBS+G, Sg PBS+G, Dual MUC5B+G	0.79	-
**Acetate (114)**	*All:* An PBS+G[I], An MUC5B+G[II], Sg PBS+G[II], Sg MUC5B+G[I], Dual PBS+G, Dual MUC5B+G	0.00031[b], 0.005[cI], 0.017[cII]	Sg MUC5B+G[I], Sg PBS+G[II]
**116**	An PBS+G, An MUC5B+G, Sg PBS+G, Dual PBS+G	0.87	-
**Alanine (118)**	*All:* An PBS+G, An MUC5B+G, Sg PBS+G, Sg MUC5B+G[I], Dual PBS+G[I], Dual MUC5B+G	0.0043[b], 0.0094[c]	Dual PBS+G
**122**	*All*	0.99	-
**Lactate (123)**	*All:* An PBS+G, An MUC5B+G[I], Sg PBS+G[I], Sg MUC5B+G, Dual PBS+G, Dual MUC5B+G	0.0005[b], 0.001[c]	Sg PBS+G
**Propionate (139)**	An PBS+G[I], Sg PBS+G, Sg MUC5B+G, Dual PBS+G, Dual MUC5B+G[I]	0.04[b], 0.02[c]	Dual MUC5B+G
**2-oxo-iso-caproate (141)**	An PBS+G[I], An MUC5B+G, Dual MUC5B+G[I]	0.02[b], 0.03[c]	An PBS+G
**143**	An PBS+G[I], Sg PBS+G, Sg MUC5B+G, Dual PBS+G, Dual MUC5B+G[I]	0.0019[b],	Dual MUC5B+G

**Butyrate (145)**	*All:* An PBS+G, An MUC5B+G, Sg PBS+G[I], Sg MUC5B+G, Dual PBS+G, Dual MUC5B+G[I]	0.006[c]<br>0.0079[b],<br>0.0028[c]	Sg PBS+G
**146**	An PBS+G, An MUC5B+G, Sg PBS+G, Sg MUC5B+G, Dual PBS+G	0.52	-
**147**	Sg PBS+G, Sg MUC5B+G, Dual PBS+G	0.68	-
**148**	*All*	0.64	-
**149**	An PBS+G, An MUC5B+G, Sg PBS+G, Sg MUC5B+G, Dual PBS+G	0.82	-
**150**	An PBS+G, An MUC5B+G, Sg PBS+G, Sg MUC5B+G, Dual PBS+G	0.17	-
**151**	*All*	0.28	-
**154**	An MUC5B+G, Sg MUC5B+G, Dual PBS+G, Dual MUC5B+G	0.65	-
